# Increasing girls’ knowledge about human papillomavirus vaccination with a pre-test and a national leaflet: a quasi-experimental study

**DOI:** 10.1186/1471-2458-13-611

**Published:** 2013-06-26

**Authors:** Robine Hofman, Puck AWH Schiffers, Jan Hendrik Richardus, Hein Raat, Inge MCM de Kok, Marjolein van Ballegooijen, Ida J Korfage

**Affiliations:** 1 Department of Public Health, Erasmus MC - University Medical Centre Rotterdam, Rotterdam, The Netherlands; 2 Institute of Health Policy and Management, Erasmus University Rotterdam, Rotterdam, The Netherlands; 3 Municipal Public Health Service Rotterdam-Rijnmond, Rotterdam, The Netherlands

**Keywords:** Human papillomavirus, Vaccination, Adolescents, Knowledge, Education

## Abstract

**Background:**

Adolescent girls are at an age to be involved in the decision about HPV vaccination uptake and therefore need adequate information about the vaccination. This study assesses to what extent reading an official information leaflet about HPV contributes to girls’ knowledge levels, and to what extent an increase in knowledge is boosted by a pre-test measurement.

**Methods:**

Participants (girls aged 11–14 years) were systematically allocated to group A that completed a pre-test measurement (12 true/false statements) or to group B that did not complete it. Subsequently, both groups read the HPV leaflet and completed the post-test measurement.

**Results:**

The response rate was 237/287 (83%). Pre-test scores in group A (M = 3.6, *SD* = 1.81, *p* < 0.001) were lower than post-test mean knowledge scores (0–10) in group B (M = 4.6, *SD* = 2.05). Post-test knowledge scores in group A were higher than those in group B [6.2 (*SD* = 2.06) versus 4.6 (*SD* = 2.05), *p* < 0.001]. In the post-test measurement, about a third of both groups knew that vaccinations do not give 100% protection against cervical cancer and that the duration of protection is unknown.

**Conclusions:**

Reading the information leaflet had a positive effect on knowledge, even more so when boosted by a pre-test measurement. However, knowledge on the degree and duration of protection against cervical cancer remained limited. Focusing girls’ attention on important aspects before they start reading the leaflet (e.g. by including a quiz on the first page) may serve to raise their awareness of these aspects.

## Background

Young adolescent girls are at an age to be involved in decisions about vaccination uptake. Countries like the United Kingdom, Canada, Australia and the Netherlands offer human papillomavirus (HPV) vaccine to girls at an age between 11 and 14 years. Girls need access to adequate information about HPV and the vaccination to be well informed about the risks/benefits of the vaccination. However, decisions about uptake are often made without sufficient information [1]. It is important that girls know, for example, that: HPV is transmitted through sexual activity and has a lifetime risk of 75-80% [2,3]; that although HPV infections are common, most infections clear within 2 years [4,5]; that an HPV infection is a necessary factor in the development of cervical cancer [6]; and that the vaccine does not provide full protection against HPV infections (it does protect against HPV 16 and 18 which are responsible for 71% of all cervical cancers [7]). Furthermore, a positive association has been found between knowledge on HPV and uptake [8,9].

Although knowledge on vaccine has been assessed among women [10-12] and adolescents [13], the impact of official information leaflets on knowledge among young adolescents has not yet been examined. This study assesses i) the extent to which girls’ knowledge levels about HPV vaccination increase after reading the official leaflet that all girls in the Netherlands receive prior to the vaccination offer, and ii) to what extent an increase in knowledge may be boosted by a pre-test measurement.

## Methods

### Participants

Girls aged 11–14 years were recruited from three secondary public schools (state funded: one urban, two rural), whilst attending their first year there. One of the authors (PAWHS) approached schools in different regions by telephone and asked if they were willing to cooperate. The number of participants was based on feasibility; however, a post-hoc power analysis showed that the power was 0.992.

### Design

In the Netherlands girls are offered the bivalent vaccine against HPV. All girls eligible for HPV vaccination receive an information leaflet about HPV and vaccination characteristics, sent by mail to their home address by the municipal health service. The leaflet includes information on how HPV is spread, the incidence of cervical cancer, the degree/duration of protection of the vaccine, the risk and symptoms of mild side-effects, and the need of a pap smear in both vaccinated and unvaccinated women. To assess girls’ knowledge levels about HPV and HPV vaccination after reading this information leaflet, we asked girls to read the leaflet (in their classroom) and to then complete a post-test measurement.

To assess the increase in girls’ knowledge levels about HPV vaccination, we needed to know the pre-reading knowledge levels and introduced a pre-test measurement. Since we acknowledged that a pre-test measurement could prompt more attentive reading of the leaflet and boost knowledge increase, a second group was introduced that did not complete a pre-test measurement. This resulted in the following design with equal numbers in both groups: girls present in the classroom were assigned to either group A (seated at one side of the classroom) which completed a pre-test measurement, then read the leaflet and immediately completed a post-test measurement; or group B (seated at the other side of the classroom) which read the leaflet and then completed the post-test measurement. There was no follow-up time between completing all the measurements and reading the leaflet.

**Figure 1:**
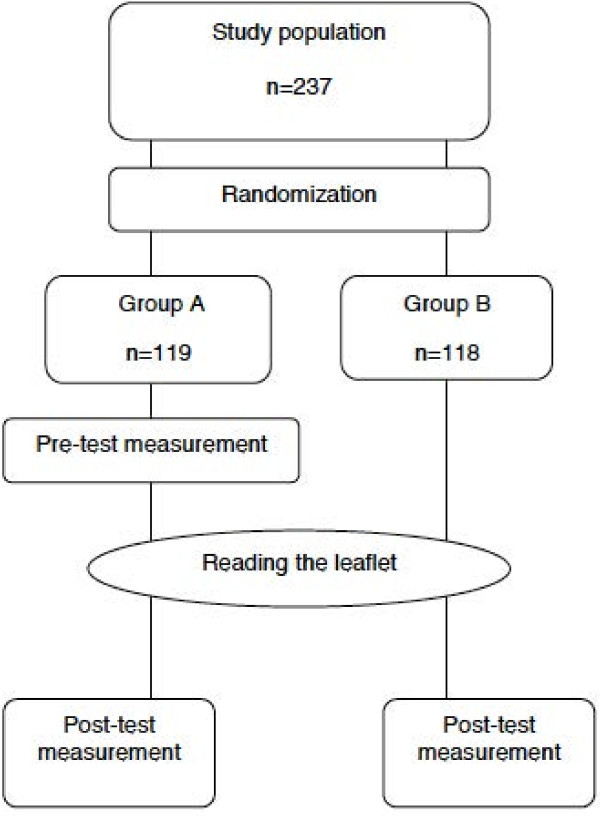
Study design in answering the two research questions.

To assess to what extent the girls’ knowledge levels about HPV vaccination increased after reading the leaflet, we compared knowledge scores of the pre-test measurement of group A with the post-test measurement of group B (Figure 1), assuming that the demographic characteristics of group A and B were similar. We hypothesized that the total knowledge score would increase after reading the leaflet.

To assess the effect of a pre-test measurement, prompting more attentive reading of the leaflet and boosting knowledge increase, we compared the post-test measurements of group A and B (Figure 1). We hypothesized that, after reading the leaflet, the total knowledge score of group A would be higher than that of group B.

It should be noted that in addressing the first research question the pre-test measurement serves as an assessment and the leaflet is interpreted as the intervention, whereas in addressing the second research question the pre-test measurement and the leaflet combined serve as the intervention (Figure 1).

### Procedure

The study was performed in accordance with the Declaration of Helsinki and was approved by the Medical Ethics Committee of Erasmus MC (MEC-2010-328). The parents of potentially participating girls received an information letter about the study and an opt-out form. Questionnaires were completed in December 2010 and January 2011 and were distributed to participants in their classrooms. A brief introduction was given on the process of completing questionnaires and reading the leaflet. Completion and reading together took 25–40 min.

### Questionnaire

The self-administered questionnaire (Additional file 1) assessed knowledge on HPV vaccination and demographic characteristics. Before presenting the questionnaire to the study population, it was piloted among three age-matched children and one teacher to evaluate its comprehensibility. Knowledge was assessed through 12 statements (Table 1). We considered eight of these statements to be essential aspects of vaccination, such as the degree/duration of protection against HPV through vaccination, and transmission of the virus. The remaining four items addressed details of the HPV vaccination, such as costs of vaccination and permission for vaccination. The correct answer to each statement could be found in the leaflet. Answer options were ‘absolutely true’, ‘possibly true’, ‘possibly not true’ and ‘absolutely not true’. We choose this response system to be able to assess respondents’ uncertainty about their answers and to assess knowledge increase at a detailed level, i.e. the percentage of respondents in group A who were not sure about their answer before reading the leaflet (marked possibly true or not true) and were sure about the correct answer after reading the leaflet (marked ‘absolutely true or not true’) (Table 1). If a statement was true the following points were assigned: absolutely true: 1 point, possibly true: 0 points, possibly not true: 0 points, and absolutely not true: 0 points. If a statement was not true, then the following points were assigned: absolutely not true: 1 point, possibly not true: 0 points, possibly true: 0 points, and absolutely true: 0 points. To facilitate interpretation of the total knowledge score, results were transformed to a 0–10 scale.

**Table 1:** Comparison of knowledge scores between group A and group B and within group A

**No.**	**Statement**	**Pre-test group A vs. post-test group B**	**Post-test group A vs. post-test group B**	**Group A: ‘almost correct’ at pre-test to ‘absolutely correct’ at post-test**
		***p-*value**		**n (%)**
1	HPV vaccinations completely protect against cervical cancer *(false).*	0.003^1a^	0.169	9 (7.6)
2	Even if you only have safe sex you can be infected with HPV *(true).*	0.001^1a^	0.079	32 (26.9)
3	All 12-year-old girls will be sent an invitation for HPV vaccinations without having to ask for it *(true).*	0.851	0.007^2^	18 (15.1)
4	Legally, parents need to give permission for HPV vaccinations in 12-year-olds *(false).*	<0.001^1a^	0.054	8 (6.7)
5	In spite of HPV vaccinations, Pap-smears from age ≥ 30 years are still recommended *(true).*	<0.001^1a^	<0.001^2^	62 (52.1)
6	You can only have a Pap smear if you have first had HPV vaccinations *(false).*	0.148	0.003^2^	16 (13.4)
7	HPV vaccinations can make you lose your hair *(false).*	<0.001^1a^	0.013^2^	40 (33.6)
8	If you have been sexually active HPV vaccinations are still advised *(true).*	0.265	0.002^2^	37 (31.1)
9	HPV vaccinations reduce the risk of getting cervical cancer *(true).*	0.006^1b^	<0.001^2^	18 (15.1)
10	We know for a fact that HPV vaccinations protect against cervical cancer for a lifetime *(false).*	0.175	0.319	18 (15.1)
11	HPV vaccinations reduce the risk of dying of cervical cancer *(true).*	0.456	<0.001^2^	26 (21.8)
12	HPV vaccinations require several hundred dollars out-of-pocket expenses *(false).*	0.131	0.999	21 (17.6)

^*1a*^ *Percentage of correct answers in the post-test measurement in group B was significantly higher compared to the pre-test measurement in group A.*

^*1b*^ *Percentage of correct answers in the post-test measurement in group B was significantly lower compared to the pre-test measurement in group A.*

^*2*^ *Percentage of correct answers in the post-test measurement in group A was significantly higher compared to the post-test measurement in group B*.

In addition, we asked girls if they were already vaccinated against HPV. If girls had not been vaccinated, we addressed their intention to get vaccinated against HPV on a 10-point Likert scale (1 = definitely not, 10 = definitely) with the following question: ‘Do you intend to get vaccinated against HPV?’

### Analyses

First, to assess whether knowledge on HPV vaccination increased after reading the leaflet, an independent samples t-test was used to analyse the difference in total knowledge scores between the pre-test measurement of group A and the post-test measurement of group B. Second, to assess to what extent an increase in knowledge was boosted by a pre-test measurement, an independent samples t-test was used to assess differences in total knowledge scores between the post-test measurements of group A and B. We assumed that pre-test knowledge levels would be similar in both groups. Cohen’s effect sizes were calculated [14]. Third, Chi-square tests were used to assess whether the number of correct answers per statement differed significantly between the pre-test measurement of group A and the post-test measurement of group B, and between the post-test measurements of both groups (Table 1). Differences between group A and B in background variables were assessed using Mann–Whitney U tests for continuous variables and Chi-square tests for categorical variables.

## Results

### Participants

The response rate was 237/287 (83%). Non-participation was due to absenteeism from school or lack of parental consent to participate. The mean age of the participants was 12.2 (*SD* group A = 0.50, *SD* group B = 0.45) years and almost all participants were born in the Netherlands (group A: 96.6%; group B: 94.9%). The majority of participants had high (group A: 41.2%; group B: 39.0%) or intermediate (group A: 34.4%; group B: 39.0%) educational level (Dutch schools have different educational levels within a school year). About half of the participants stated they had a religious affiliation (group A: 55.6%; group B: 50%). Group A and B showed no significant differences regarding demographic characteristics and HPV vaccination history (Table 2).

**Table 2:** Characteristics of the study participants

**Characteristics**	**Group A**		**Group B**		
	**(n = 119)**		**(n = 118)**		***p*-value**
	**Mean**	**(SD)**	**Mean**	**(SD)**	
Age (years)	(12.2)	(0.50)	(12.2)	(0.45)	0.82
Age range (years)	11-14		11-13		
	n	(%)	n	(%)	
**Educational level**					0.76
Low	29	(24.4)	26	(22.0)	
Intermediate	41	(34.4)	46	(39.0)	
High	49	(41.2)	46	(39.0)	
**Religion**					0.40
None	52	(44.4)	59	(50.0)	
Christian	64	(54.7)	56	(47.5)	
Islam	1	(0.9)	1	(0.8)	
Other	0	(0.0)	2	(1.7)	
**Country of birth of participants**					0.74
The Netherlands	115	(96.6)	112	(94.9)	
**Country of birth of parents**					0.75
Both parents born in the Netherlands	102	(86.4)	97	(86.6)	
One parent born outside the Netherlands	9	(7.6)	9	(8.0)	
Both parents born outside the Netherlands	7	(5.9)	6	(5.4)	
**HPV vaccinated before completion of questionnaire**					
Yes	22	(18.5)	17	(14.5)	0.52
**Intention if not vaccinated**					0.26
Low	12	(12.5)	21	(21)	
Neutral	18	(18.8)	19	(19)	
High	66	(68.7)	60	(60)	

Note: Group A and B had no significant differences regarding demographic characteristics and HPV vaccination history.

### Comparison of knowledge scores before and after reading the leaflet

**Figure 2:**
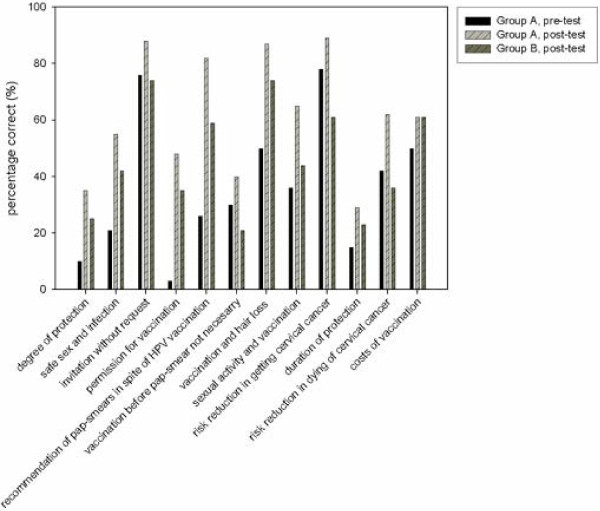
Percentage of correct answers to the statements made by group A and B.

For these analyses, total knowledge scores of the pre-test measurement of group A (n = 119) were compared with the scores of the post-test measurement of group B (n = 118). As hypothesized, we found that total knowledge scores were significantly lower in group A before reading the leaflet (M = 3.6, SD = 1.81) than in group B that completed the questionnaire after (M = 4.6, SD = 2.05) reading the leaflet, t(235) = -3.941, *p <* 0.001. Cohen’s effect size was 0.52, indicating a moderate effect [14]. Figure 2 shows the distribution of correct answers per knowledge statement about HPV and cervical cancer.

The number of correct answers to 5 of 12 statements was significantly lower in group A (n = 119) before reading the leaflet than in group B (n = 118) after reading the leaflet. For instance, statement 2 about safe sex and infection [group A: 25/119 (21.0%), group B: 50/118 (42.4%); *p* = 0.001], statement 1 about incomplete protection against cervical cancer [group A: 12/119 (10.1%), group B: 30/118 (25.4%); *p* = 0.003], and statement 10 about unknown duration of protection against cervical cancer [group A: 18/119 (15.1%), group B: 27/118 (22.9%); *p* = 0.175]. However, statement 9 about the risk reduction of getting cervical cancer after being vaccinated was answered correctly less often by group B after reading the leaflet than by group A before reading the leaflet (pre: 78.2%, post: 61.0%; *p* = 0.006) (Figure 2) (Table 1).

We assessed the number of respondents in group A who had an ‘almost correct’ answer before reading the leaflet and an ‘absolutely correct’ answer after reading the leaflet. Respondents were most reassured by the leaflet about the correct answer considering the following statements: statement 5 about the recommendation of pap smears in spite of HPV vaccination 62/119 (52.1%); statement 7 about hair loss after vaccination 40/119 (33.6%); and statement 8 about sexual activity and vaccination 37/119 (31.1%) (Table 1).

### Influence of pre-test measurement on knowledge scores at post-test measurement

Comparing knowledge scores of both post-tests between group A and B showed, as hypothesized, that group A (n = 119) (*M* = 6.2, *SD* = 2.06) had a significantly higher total knowledge score at the post-test measurement than group B (n = 118) (*M* = 4.6, *SD* = 2.05), *t*(235) = 5.805, *p* < 0.001). Cohen’s effect size was 0.78, indicating a moderate effect [14]. After completing a pre-test measurement and reading the leaflet (group A), the number of correct answers to 7 of 12 statements was significantly larger than after reading the leaflet only (group B) (Table 1).

## Discussion

This study examined the knowledge among girls aged 11–14 years about HPV and vaccination, and the extent of increase in knowledge after reading the official HPV vaccination leaflet used in the Dutch national immunization program. Firstly, total knowledge scores were higher after reading the information leaflet and we conclude that reading it had a positive effect on the knowledge levels of the girls. Secondly, post-leaflet total knowledge scores were higher in girls who had also completed the questionnaire *before* reading the leaflet and we conclude that completing this questionnaire had a positive effect on the knowledge levels.

Inclusion of a second group that did not complete a pre-test allowed to assess the effect of a pre-test measurement on knowledge scores. The characteristics of both groups were similar, indicating that systematically dividing the girls into two groups worked well and the groups were comparable. The higher post-leaflet knowledge scores in girls who had also completed the questionnaire before reading the leaflet are probably due to the girls’ attention being prompted by the statements in the questionnaire, and their increased awareness of the knowledge they were supposed to have at the post-test measurement. This may have led to more attentive reading of the leaflet and thus being able to answer more statements correctly. Such a booster effect of a pre-test measurement, in fact acting as an intervention, is called the mere measurement effect [15]. This effect was also found in a study among novice blood donors; people who completed a questionnaire about blood donation were more willing to give blood than those who had not completed a questionnaire [15].

The percentage of correct answers to some statements largely increased from a low percentage before reading the leaflet to a high percentage after reading the leaflet, e.g. the statements about whether girls need permission from their parents to get vaccinated and that, despite HPV vaccinations, pap smears are still recommended. The leaflet had a positive effect on increased knowledge scores after reading it. Because some statements were already answered correctly by most girls before reading the leaflet, there was less room for improvement in knowledge. Surprisingly, knowledge on the degree/duration of protection against cervical cancer was low before reading the leaflet and remained relatively low after reading it. For instance, about 75% of the girls incorrectly thought that vaccination completely protects against cervical cancer and that protection lasts a lifetime. For optimal benefit from HPV vaccination, girls need to know that booster vaccinations might be needed in the future and that other preventive measures, such as screening, are still recommended. We advise additional education about the recommendation to participate in cervical cancer screening also after HPV vaccination. The group who completed the statements before *and* after reading the leaflet had better knowledge scores at the post-test measurement regarding all statements. With the exception of one statement, knowledge on the risk reduction of getting cervical cancer after HPV vaccination was worse *after* reading the leaflet in one group than before reading the leaflet in the other group. A possible explanation for this might be that girls who completed the pre-test measurement were better informed about HPV vaccination before completing the pre-test and reading the leaflet; however, their knowledge on this item increased after reading the leaflet. For this reason, we suggest that this specific item be thoroughly revised when the leaflet is e.g. updated.

We acknowledge that it is preferable to use larger groups, and to randomise in a more sophisticated way than simply dividing one side of the classroom from the other. Overall, to improve girls’ understanding of the purpose of vaccination and the degree/duration of protection against cervical cancer, we recommend that information be unambiguous and that the key points should be clearly outlined on a prioritized list [16]. This can be achieved by, e.g., editing or improving the current leaflet, or offering information on these important aspects at school or other relevant locations.

A limitation is that we only have data on the girls’ intention to have (or not have) the vaccination, and lack information on the actual decision about uptake. Strengths of the study are its external validity: the use of an official leaflet which is sent to every 12-year-old girl in the Netherlands, the high response rate (83%), and the fact that the leaflet addresses a choice that participants have to make in real life. However, reading the leaflet at school is different from reading it at home and, due to non-probability sampling; the results may not represent the entire population.

## Conclusion

This study shows that reading the information leaflet had a positive effect on girls’ knowledge about HPV, which showed a further increase when boosted by a pre-test measurement. However, levels of knowledge regarding the degree/duration of protection against cervical cancer remained low. Prompting girls’ attention before they start reading the leaflet may raise their awareness of important aspects of HPV vaccination and may give better support in their decision-making process. This could, for example, be organized by conducting a quiz at school, by including a quiz on the first page of the leaflet, or by conducting a quiz on the internet which has the advantage of being able to provide tailored information based on a girl’s knowledge score.

## Competing interest

The authors declare that they have no competing interests.

## Authors’ contributions

IJK conceived the idea for the study, designed the protocol and supervised the performance of the study; All authors contributed to the design of the questionnaire; PAWHS performed the retrieval of the sample; PAWHS was responsible for the database design and data entry, and performed the preliminary analyses; RH performed the final analyses; IJK, RH, JHR, HR, IMCMdK and MvB discussed the interpretation of the results; PAWHS drafted a preliminary report and RH drafted a final report; all authors revised the article critically. All authors read and approved the final manuscript.

## Pre-publication history

The pre-publication history for this paper can be accessed here:

http://www.biomedcentral.com/1471-2458/13/611/prepub

## Supplementary Material

Additional file 1 Questionnaire, word document. Click here for file
