# Supplementary material for: Increasing girls’ knowledge about human papillomavirus vaccination with a pre-test and a national leaflet: a quasi-experimental study
Source: BMC Public Health. 2013 Jun 26;13:611. doi: 10.1186/1471-2458-13-611 (PMC3698036; doi:10.1186/1471-2458-13-611)
Supplement: Additional file 1 — Questionnaire, word document. [file 1471-2458-13-611-S1.doc]

Additional file 1 – Questionnaire

|  |  | **Absolutely true** | **Possibly true** | **Possibly not true** | **Absolutely not true** |
| --- | --- | --- | --- | --- | --- |
| 1 | HPV vaccinations completely protect against cervical cancer | **🞏** | **🞏** | **🞏** | **🞏** |
| 2 | Even if you only have safe sex you can be infected with HPV | **🞏** | **🞏** | **🞏** | **🞏** |
| 3 | All 12-year-old girls will be sent an invitation for HPV vaccinations without having to ask for it | **🞏** | **🞏** | **🞏** | **🞏** |
| 4 | Legally, parents need to give permission for HPV vaccinations in 12-year-olds | **🞏** | **🞏** | **🞏** | **🞏** |
| 5 | In spite of HPV vaccinations, Pap-smears from age ≥ 30 years are still recommended*.* | **🞏** | **🞏** | **🞏** | **🞏** |
| 6 | You can only have a Pap smear if you have first had HPV vaccinations | **🞏** | **🞏** | **🞏** | **🞏** |
| 7 | HPV vaccinations can make you lose your hair | **🞏** | **🞏** | **🞏** | **🞏** |
| 8 | If you have been sexually active HPV vaccinations are still advised | **🞏** | **🞏** | **🞏** | **🞏** |
| 9 | HPV vaccinations reduce the risk of getting cervical cancer | **🞏** | **🞏** | **🞏** | **🞏** |
| 10 | We know for a fact that HPV vaccinations protect against cervical cancer for a lifetime | **🞏** | **🞏** | **🞏** | **🞏** |
| 11 | HPV vaccinations reduce the risk of dying of cervical cancer | **🞏** | **🞏** | **🞏** | **🞏** |
| 12 | HPV vaccinations require several hundred dollars out-of-pocket expenses | **🞏** | **🞏** | **🞏** | **🞏** |

| **Are you already vaccinated against HPV?** |
| --- |

|  | **🞏** | No |
| --- | --- | --- |
|  | **🞏** | Yes, 1 vaccination |
|  | **🞏** | Yes, 2 vaccinations |
|  | **🞏** | Yes, 3 vaccinations |

| **Do you intend to get vaccinated against HPV?** | | | | | | | | | |
| --- | --- | --- | --- | --- | --- | --- | --- | --- | --- |
| Definitely not |  |  |  |  |  |  |  |  | Definitely |
| **1** | **2** | **3** | **4** | **5** | **6** | **7** | **8** | **9** | **10** |

| **Have you read the information leaflet previously?** |
| --- |

|  | **🞏** | Yes |
| --- | --- | --- |
|  | **🞏** | No |

| **Age:…….years** |
| --- |

| **Educational level** |
| --- |

|  | **🞏** | Low |
| --- | --- | --- |
|  | **🞏** | Intermediate |
|  | **🞏** | High |

| **Religion** |
| --- |

|  | **🞏** | None |
| --- | --- | --- |
|  | **🞏** | Christianity |
|  | **🞏** | Islam |
|  | **🞏** | Hinduism |
|  | **🞏** | Other:……. |

| **Country of birth** | | | |
| --- | --- | --- | --- |
|  | **a. you** | **b. your father** | **c. your mother** |
| The Netherlands | **🞏** | **🞏** | **🞏** |
|  |  |  |  |
| Outside the Netherlands | **🞏** | **🞏** | **🞏** |
|  |  |  |  |
